# HOMA-beta independently predicts survival in patients with advanced cancer on treatment with immune checkpoint inhibitors

**DOI:** 10.3389/fendo.2024.1439705

**Published:** 2024-12-11

**Authors:** Mayu Watanabe, Jun Eguchi, Atsushi Takamoto, Hiromitsu Kanzaki, Yohei Noda, Syunsuke Kagawa, Jun Wada

**Affiliations:** ^1^ Department of Nephrology, Rheumatology, Endocrinology and Metabolism, Okayama University Graduate School of Medicine, Dentistry and Pharmaceutical Sciences, Okayama, Japan; ^2^ Department of Diabetology and Metabolism, NHO Okayama Medical Center, Okayama, Japan; ^3^ Department of Urology, Fukuyama City Hospital, Hiroshima, Japan; ^4^ Department of Internal Medicine, Tsuyama Chuo Hospital, Okayama, Japan; ^5^ Department of Gastroenterological Surgery, Okayama University Graduate School of Medicine, Dentistry and Pharmaceutical Sciences, Okayama, Japan; ^6^ Clinical Cancer Center, Okayama University Hospital, Okayama, Japan

**Keywords:** anti-PD1 immune checkpoint inhibitors, insulin secretory capacity, cancer prognosis, insulin secretion, glucose metabolism markers

## Abstract

**Background:**

Although immune checkpoint inhibitors (ICIs) are effective cancer drugs, ICI-induced diabetes is a rare but a life-threatening adverse event for patients. The deleterious action of ICI on pancreatic beta-cell function is a concern. However, the influence of ICI on insulin synthesis and secretion in patients with cancer without diabetes remains unknown.

**Methods:**

This study included 87 patients diagnosed with advanced cancer. Glucose metabolism markers (HbA1c, HOMA-IR) and indicators of insulin secretory capacity (HOMA-beta, C-peptide) were prospectively evaluated in patients with ICI-treated cancers to determine their association with cancer prognosis.

**Results:**

Patients with overall survival (OS) ≥ 7 months had substantially higher HOMA-beta levels at baseline (p=0.008) and 1 month after ICI administration (p=0.006) compared to those with OS <7 months. The median OS was significantly longer in patients with HOMA-beta ≥ 64.24 (13 months, 95%CI: 5.849–20.151, 37 events) than in those with HOMA-beta < 64.24 (5 months, 95%CI: 3.280–6.720, 50 events) (p=0.013). Further, the median progression-free survival (PFS) was significantly longer in patients with HOMA-beta ≥ 66.43 (4 months, 95%CI: 3.073–4.927, 33 events) than in those with HOMA-beta < 66.43 (2 months, 95%CI: 1.410–2.590, 54 events) (p=0.025). Additionally, multivariable logistic regression analysis revealed that a HOMA-beta value ≥ 64.24 independently predicted longer OS in ICI-treated patients.

**Conclusions:**

Pre-ICI HOMA-beta level is linked to longer OS in ICI-treated patients. This connection is significant and shows that insulin secretory capacity may predict ICI efficacy.

## Introduction

The use of immune checkpoint inhibitors (ICIs) has significantly expanded as a highly efficacious anticancer approach for several types of malignancies (1). Timely identification and effective handling of immune-related adverse events (irAEs) are crucial for mitigating the substantial morbidity arising from the growing utilization of immunotherapeutic interventions (2). The incidence of immune checkpoint inhibitor-induced diabetes mellitus (CPI-DM) among irAEs is rare, less than 1%; however, it is a life-threatening adverse effect that poses a significant risk to a patient’s life, with no means of early detection (3, 4). Previous studies have reported several features of CPI-DM: rapid onset of severe hyperglycemia, the presence of diabetic ketoacidosis or an extreme reduction in C-peptide levels, and continuous insulin dependence for glycemic control following the development of diabetes (5). Moreover, Hisanaga et al. observed that pre-existing diabetes is associated with higher mortality in advanced lung cancer during ICI treatment (6). Matsumura et al. documented that nivolumab administration resulted in the exacerbation of diabetes and partial insulin secretion deficiency in patients diagnosed with lung cancer (7). Although several studies have reported the development of insulin-deficient diabetes mellitus after ICI administration, it is not necessarily a type of insulin-depleted diabetes (3, 4). The potential impact of ICI administration on pancreatic beta-cell activity has been a subject of investigation. However, limited information is available on the effects of ICI treatment on insulin secretion in individuals without diabetes. Gauci et al. retrospectively investigated the impact of ICI administration on blood glucose levels in a cohort of 116 patients, excluding those with CPI-DM. Their findings revealed no statistically significant alterations in blood glucose levels following ICI administration. Furthermore, there is a lack of research investigating the impact of ICI administration on C-peptide, HOMA-beta, and HOMA-IR (8). This prospective observational study was conducted to analyze the impact of ICI administration on indicators of glucose metabolism in cancer patients without diabetes. Additionally, this study aimed to explore the potential association between glucose metabolism markers and the efficacy of ICI.

## Materials and methods

### Study participants

The present study included a cohort of 87 individuals diagnosed with advanced cancer who were recruited between June 2017 and August 2019. Advanced cancer was defined as a case in which the cancer was histologically stage IV and had progressed or recurred during or after one or more systemic treatments for advanced or metastatic disease. The study included patients who were diagnosed with stage IV cancer, as confirmed via histological examination, and were recruited from Okayama University Hospital. The exclusion criteria were as follows: (1) a documented medical history of diabetes; (2) fasting blood glucose levels ≥ 126 mg/dL (7.0 mmol/L) or glycated hemoglobin (HbA1c) values ≥ 6.5%; and (3) treatment with anti-diabetes medication. All participants were administered a minimum of one dose of intravenous anti-PD1 drug as initial immunotherapy. Participants in this study did not receive concomitant steroid therapy or chemotherapy during the observation period. Prognosis was monitored until September 2022. Written informed consent was obtained from all patients. The study protocol was approved by the Ethics Committee of Okayama University (1704-009) and was conducted in accordance with the Declaration of Helsinki.

### Data collection

Blood samples were collected after a 12-h fast. HbA1c, fasting plasma glucose (FPG), C-reactive protein, and creatinine levels were measured within 1 h of blood collection using standard methods and an automated clinical chemistry analyzer (JCA-BM8040G, JEOL Ltd, Tokyo, Japan). Fasting serum C-peptide (Lumipulse System, Fujirebio, Tokyo, Japan) and immunoreactive insulin (IRI) (Chemilumi Insulin, Siemens Healthineers, Tokyo, Japan) levels were measured.

The body mass index (BMI) was calculated using the following formula: body weight (kg)/height^2^ (m^2^). The homeostasis model of assessment-insulin resistance (HOMA-IR) was calculated using the following formula: fasting insulin (μU/mL) × fasting glucose (mg/dL)/405. The homeostasis model of assessment-beta cell (HOMA-beta) was calculated as follows: 360 × fasting insulin (μU/mL)/(fasting glucose (mg/dL) − 63). eGFR was calculated using the following formula modified for Japanese subjects: eGFR (mL/min/1.73 m^2^) = 194 × s-Cr (mg/dL)^-1.094^ × age (years) ^-0.287^ (× 0.739, for females).

Medical history and current status of primary tumor progression, Eastern Cooperative Oncology Group performance status (ECOG-PS; 0–1 vs. ≥2), and number of metastatic sites were collected from the medical charts of each patient.

### Definition

The response to ICI therapy was assessed by evaluating the overall survival (OS) and progression-free survival (PFS) using the Response Evaluation Criteria in Solid Tumors (version 1.1) (9).

OS was defined as the time from the initiation of the first ICI administration to death. PFS was defined as the time from initiation of the first ICI administration to disease progression or death. PFS was determined for patients who did not exhibit disease progression as of September 2022 and was defined as the date of the most recent imaging examination. The cohort of patients who remained alive as of September 2022 was determined based on the time from therapy initiation to the date of their most recent visit.

### Statistical analysis

The paired Wilcoxon signed-rank and Friedman tests were performed to evaluate the changes in glucose tolerance before and after ICI administration. The Mann–Whitney U test was used to compare the two groups based on the median overall survival. Receiver operating characteristic (ROC) curves were calculated for glucose metabolism markers (BMI and HOMA-beta) to define the cut-off value by the method with the smallest distance from the point in the upper left corner in two groups with median OS. PFS and OS from the date of the first treatment and survival curves were generated using the Kaplan–Meier method and compared using the log-rank test. Univariate and multivariate Cox proportional hazards models were used to calculate the independent significance of prognostic variables. All statistical analyses were performed using SPSS version 26 (IBM Corp., Armonk, NY, USA). Statistical significance was set at p<0.05.

## Results

### Baseline characteristics of the study participants

This study included 87 participants (59 male and 28 female). The baseline characteristics of the study participants are presented in **Table 1**. The median age of the participants was 65 years, with a median BMI of 19.2 kg/m^2^. Of the participants, 78 individuals used nivolumab, 10 individuals used pembrolizmab, and 1 individual used ipilimumab. Regarding the combination therapy, a single patient received a treatment regimen consisting of nivolumab and pembrolizmab, while another patient received nivolumab in conjunction of ipilimumab. Additionally, the Eastern Cooperative Oncology Group Performance Status (ECOG-PS) was classified as 0-1 in 80.5% of the cases. We observed a total of 52 cases of head and neck cancer, 19 cases of gastric cancer, and 16 cases of various other types of cancer. Specifically, these included six cases of renal cell carcinoma, six cases of urothelial cancer, two cases of colon cancer, one case of cervical esophageal cancer, and one case of malignant melanoma. The laboratory results showed no indications of renal dysfunction or overt diabetes among the individuals.

**Table 1:** The baseline characteristics of the study participants.

	All patients (n=87)
Age (years)	65 (56–72)
Sex (Male)	59 (67.8)
BMI (kg/m^2^)	19.2 (17.7–21.4)
Primary Tumor (%)	
Head and Neck cancer	52 (59.8)
Gastric cancer	19 (21.3)
Other	16 (18.3)
ICI (%)	
Nivolumab	78 (89.7)
Pembrolizumab	11 (12.6)
Ipilimumab	2 (2.3)
ECOG-PS	
0–1	70 (80.5)
≥2	16 (18.4)
Laboratory data	
CRP (mg/dL)	0.425 (0.19–3.32)
Cr (mg/dL)	0.75 (0.65–0.90)
eGFR (mL/min/1.73m^2^)	70.9 (63.5–87.2)
Glucose metabolism markers	
HbA1c (%)	5.6 (5.3–5.9)
Fasting plasma glucose (mg/dL)	97 (88–110)
C-peptide (ng/mL)	1.52 (1.01–2.24)
IRI (IU/mL)	5.2 (3.2–8.7)
HOMA-beta	59.4 (37.1–85.3)
HOMA-IR	1.11 (0.72–2.34)

Data are shown as median (25–75th percentile) for continuous variables and as percentages for categorical variables.

BMI, body mass index; ICI, immune checkpoint inhibitor; CRP, C-reactive protein; HbA1c, glycated hemoglobin; HOMA-beta, homeostasis model assessment of beta-cells; HOMA-IR, homeostasis model assessment of insulin resistance.

### Glucose metabolism markers after ICI administration

**Figure 1:**
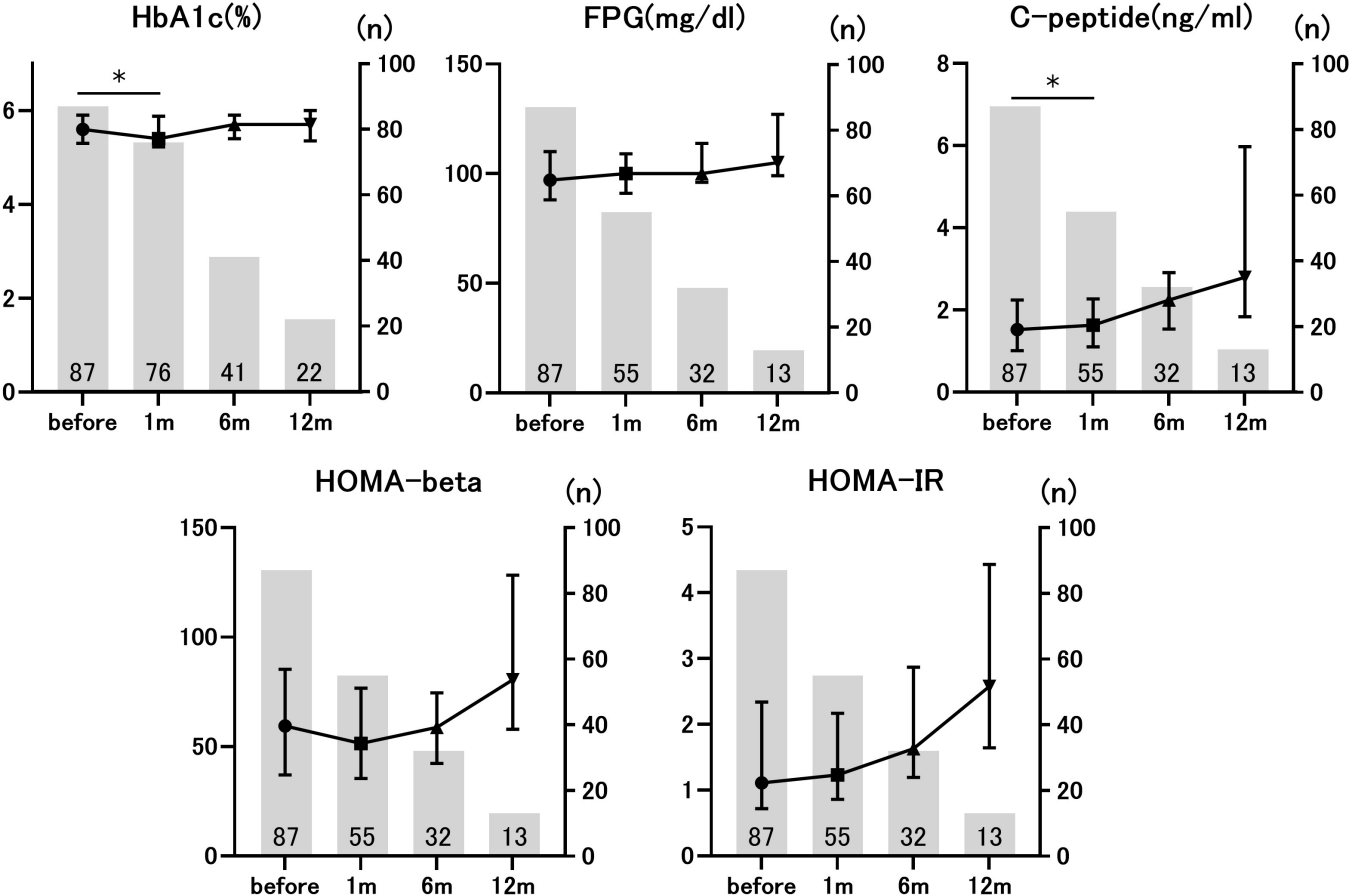
Longitudinal changes in glucose metabolism markers before and after immune checkpoint inhibitors (ICIs) administration. The number of patients applicable to each time point is given as a numerical value. *p<0.05 vs. pre-ICI treatment. Data are presented as mean ± SD.

We evaluated the time course of the glucose metabolism markers before and after ICI administration. HbA1c levels before and 1 month after ICI administration were compared in 76 individuals. The median HbA1c level before ICI administration was 5.6% (interquartile range [IQR], 0.58), whereas the median HbA1c level 1 month after ICI administration was 5.4% (IQR, 0.65). This difference was statistically significant (p=0.018). Similarly, C-peptide levels at baseline (1.51 ng/mL [IQR 1.06]) significantly increased 1 month after ICI administration (1.63 ng/mL [IQR 1.17]) (p=0.022, n=55). However, no statistically significant changes were observed in FPG, HOMA-beta, or HOMA-IR (**Figure 1**). The number of evaluable patients gradually decreased over the course of 6 months as ICI were deemed ineffective, and ICI therapy was discontinued. There were no statistically significant differences in glucose metabolism markers between the 14 patients who were evaluated at 6 months and the 5 patients who were evaluated at 12 months after ICI administration. Since the analysis was performed in participants without diabetes, the ICI-induced changes in HbA1c and C-peptide were not clinically meaningful, but these data suggested that ICI administration affected glucose metabolism markers even in non-diabetic conditions.

### Glucose metabolism markers and overall survival

**Figure 2:**
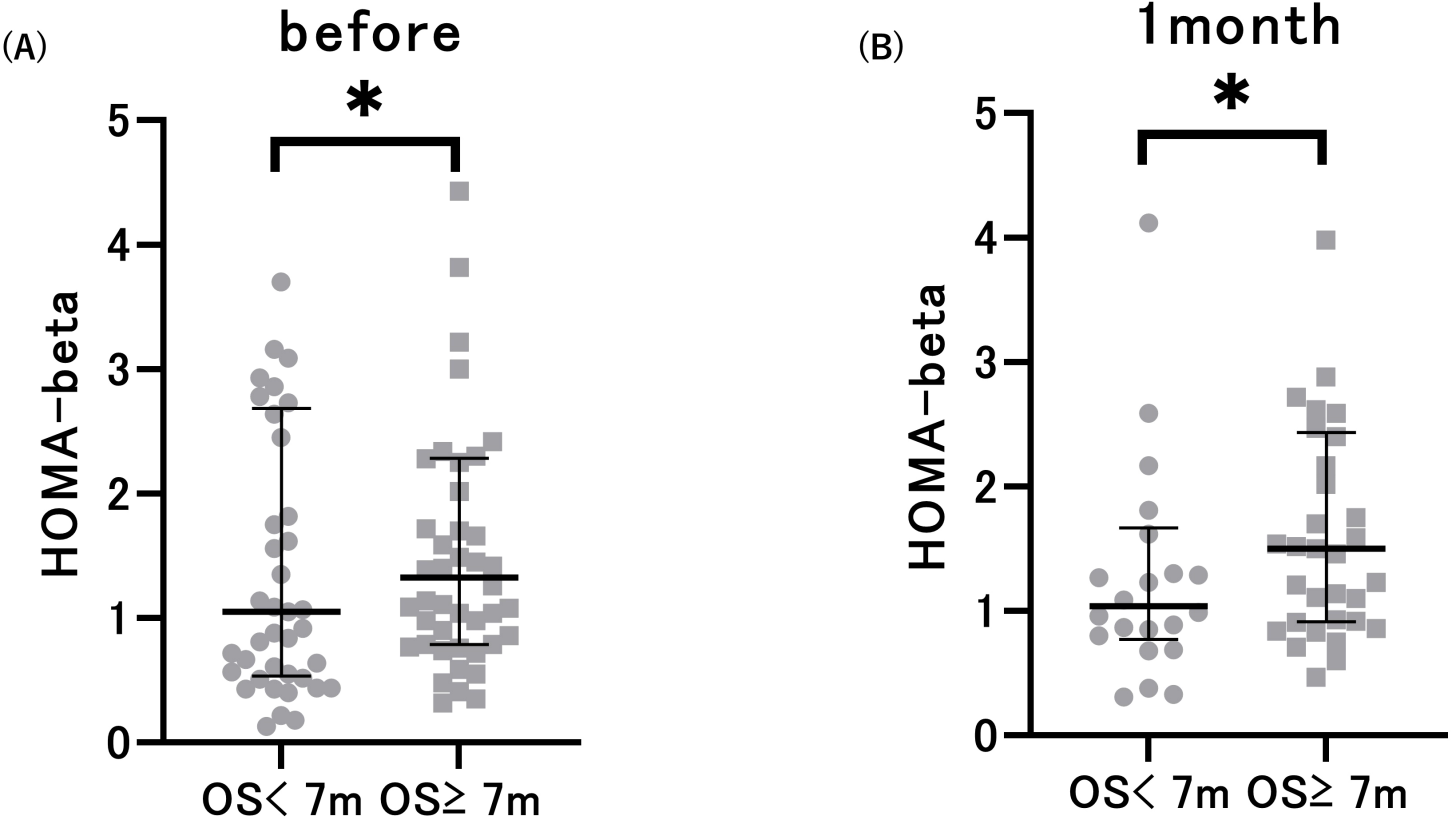
Pre-treatment HOMA-beta **(A)** and HOMA-beta after 1 month of treatment **(B)** in patients with overall survival (OS) < 7 months and OS ≥ 7 months. *p<0.05 vs. patients with OS < 7 months. Data are presented as mean ± SD.

Next, we compared glucose metabolism markers based on the median OS. The median OS was 7 months (95%CI: 4.892–9.108). We observed a substantial decrease in HbA1c levels 1 month after ICI treatment in patients with an OS of at least 7 months compared to their pre-treatment levels. Serum C-peptide levels significantly decreased after 1 month of therapy in patients with an OS of less than 7 months (**Table 2**). Notably, patients with OS greater than 7 months exhibited increased pre-treatment HOMA-beta and HOMA-beta after 1 month of treatment, in comparison to patients with OS less than 7 months (**Figure 2**). This difference was observed both before (p=0.008) and 1 month after (p=0.006) ICI administration. However, there were no significant differences in HbA1c, FPG, C-peptide, IRI, or HOMA-IR. These data suggest an association between the pre-ICI HOMA-beta and OS.

**Table 2:** Comparison of glucose metabolism markers before and 1 month after ICI administration in the two groups based on median overall survival (OS).

	OS < 7months		P-value	OS ≥ 7months		P-value
	before	1month		before	1month	
HbA1c (%)	5.6 (0.65)	5.45 (0.53)	0.317	5.6 (0.50)	5.40 (0.75)	0.027
FPG (mg/dL)	100 (28.5)	103.5 (28.3)	0.513	94.0 (17.0)	100 (13.5)	0.157
C-peptide (ng/mL)	1.89 (1.51)	1.59 (1.36)	0.033	1.50 (0.57)	1.63 (0.91)	0.223
IRI (IU/mL)	4.25 (6.6)	4.20 (2.7)	0.827	5.6 (3.8)	5.9 (5.0)	0.117
HOMA-beta	57.0 (38.1)	40.9 (34.7)	0.201	65.9 (53.3)	61.3 (50.6)	0.862
HOMA-IR	1.11 (2.03)	1.04 (0.89)	1.000	1.26 (0.88)	1.50 (1.52)	0.223

FPG, fasting plasma glucose; IRI, immunoreactive insulin; OS, overall survival; ICI, immune checkpoint inhibitor; HbA1c, glycated hemoglobin; HOMA-beta, homeostasis model assessment of beta-cells; HOMA-IR, homeostasis model assessment of insulin resistance.

### Association of glucose metabolism markers with cancer prognosis

**Figure 3:**
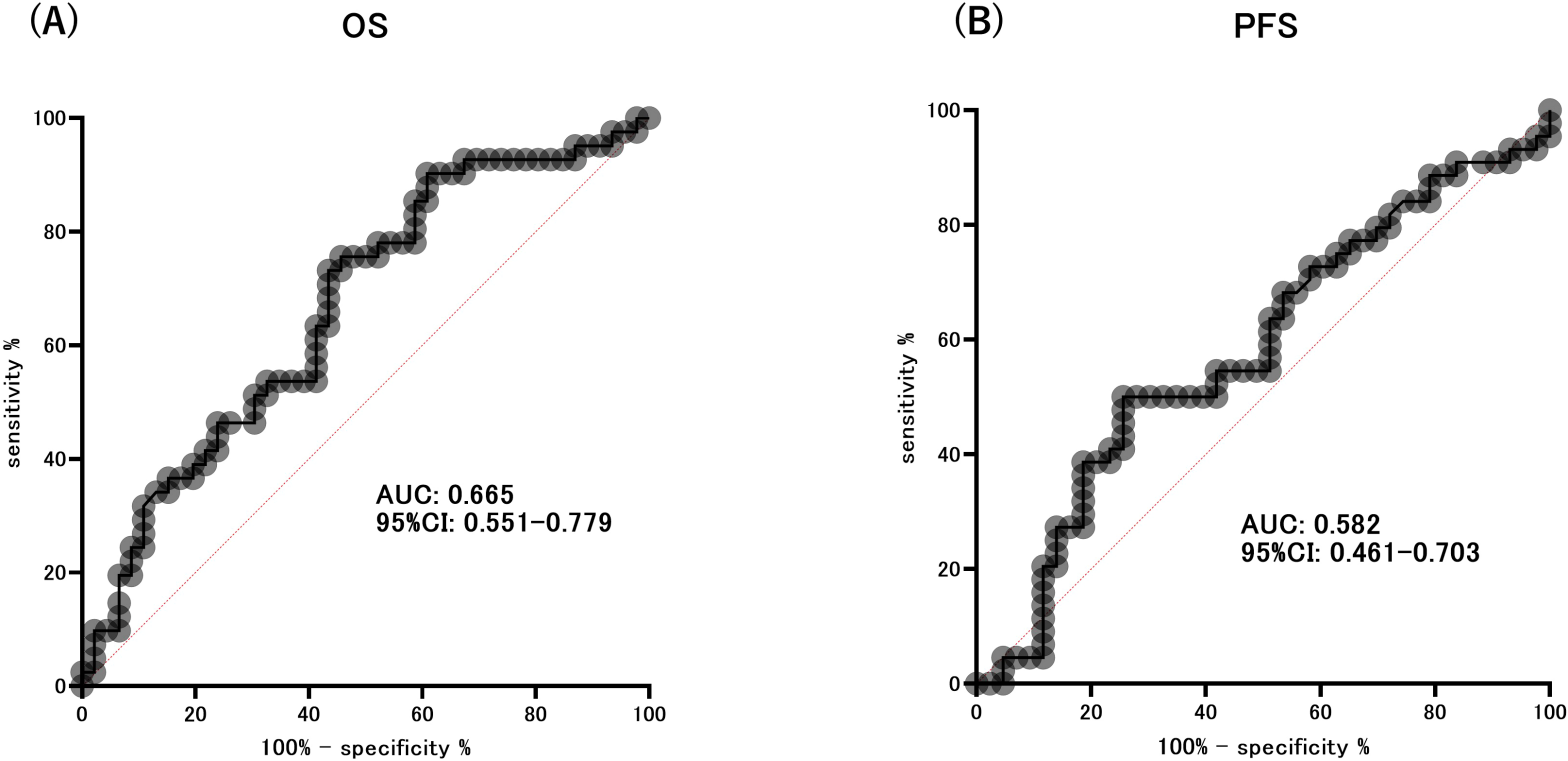
ROC curve analysis of the ability of HOMA-beta to predict the extended overall survival (OS) **(A)** and progression-free survival (PFS) **(B)**.

**Figure 4:**
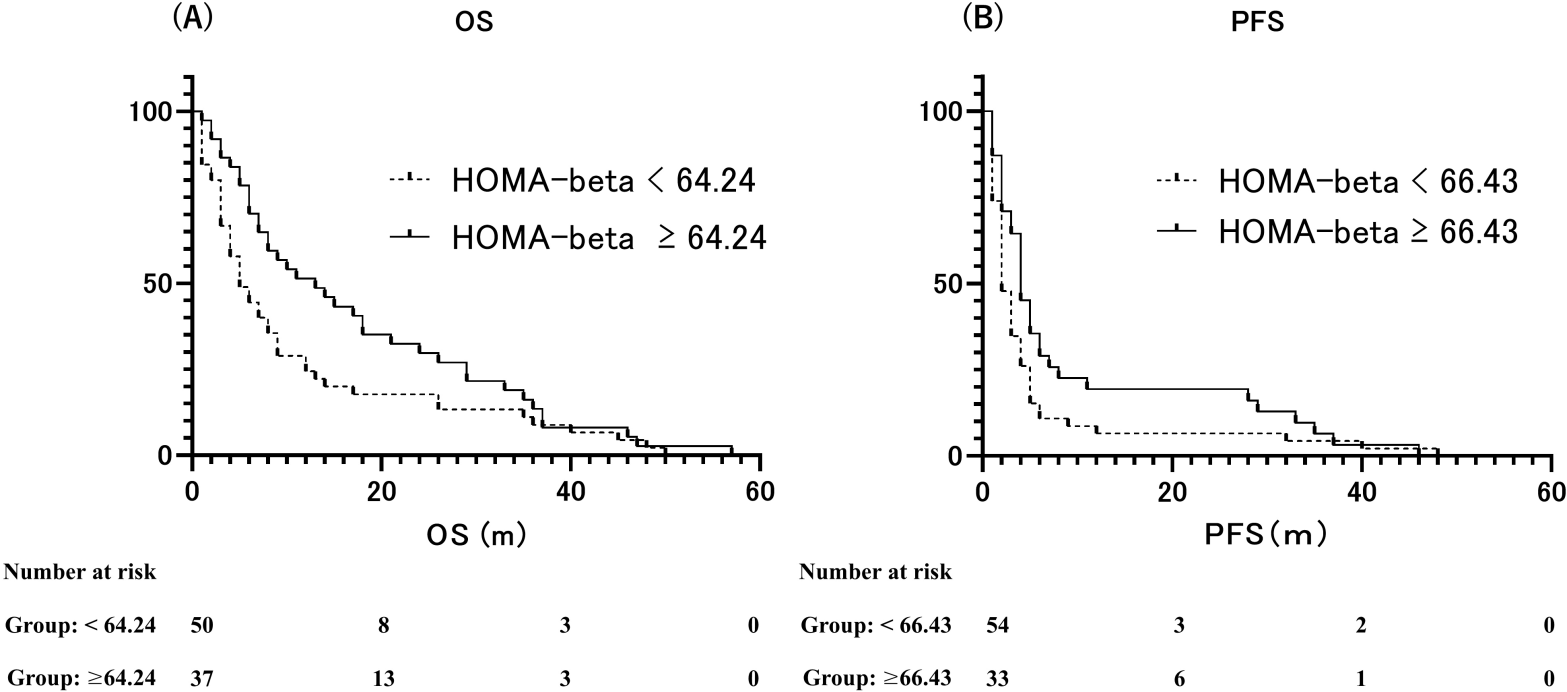
**(A)** Kaplan–Meier curves of overall survival (OS) for patients with HOMA-beta ≥ 64.24 and HOMA-beta < 64.24. **(B)** Kaplan–Meier curves of progression-free survival (PFS) for patients with HOMA-beta ≥ 66.43 and HOMA-beta < 66.43.

At the end of the observation period, disease progression was observed in 75 individuals, accounting for 86.2% of the total participants. Additionally, the mortality rate was 94.3%, with 82 patients succumbing to the condition. The median OS was 7 months (95% CI: 4.892–9.108 months), and the median PFS was 3 months (95% CI: 2.247–3.753 months). We assessed the ability of the area under the ROC curve based on BMI and HOMA-beta to predict the median OS (OS ≥ 7 months) in patients with ICI treatment. The optimal predictive BMI cut-off value was found to be 18.58 kg/m^2^ with a sensitivity of 0.717, specificity of 0.561, and AUC of 0.665 (p=0.008, **Supplementary Figure S1A**). HOMA-beta cut-off value was 64.24, with a sensitivity of 0.565, specificity of 0.732, and AUC of 0.665 (p=0.008, **Figure 3A**). Moreover, we evaluated the ability of the area under the ROC curve based on BMI and HOMA-beta to predict the median PFS (PFS ≥ 3 months) in patients undergoing ICI treatment. The optimal predictive BMI cut-off value was 18.44 kg/m^2^, with a sensitivity of 0.727, specificity of 0.512, and AUC of 0.631 (p=0.035, **Supplementary Figure S1B**). The HOMA-beta cut-off value was 66.43, with a sensitivity of 0.500, specificity of 0.744, and AUC of 0.582 (p=0.185, **Figure 3B**). The median OS was significantly longer in patients with BMI ≥ 18.58 kg/m^2^ (12 months, 95%CI: 7.633–16.367, 51 events) than in those with BMI < 18.58 kg/m^2^ (5 months, 95% CI: 3.343–6.657, 36 events) (p=0.001; **Supplementary Figure S2A**). Additionally, the median OS was significantly longer in patients with HOMA-beta ≥ 64.24 (13 months, 95%CI: 5.849–20.151, 37 events) than in those with HOMA-beta < 64.24 (5 months, 95%CI: 3.280–6.720, 50 events) (p=0.013; **Figure 4A**). Further, the median PFS was significantly longer in patients with HOMA-beta ≥ 66.43 (4 months, 95%CI: 3.073–4.927, 33 events) than in those with HOMA-beta < 66.43 (2 months, 95%CI: 1.410–2.590, 54 events) (p=0.025; **Figure 4B**). The median PFS tended to be longer in patients with BMI ≥ 18.44 kg/m^2^ (4 months, 95%CI: 2.866-5.134, 53 events) than in those with BMI < 18.44 kg/m^2^ (2 months, 95% CI: 1.454-2.546, 34 events), but the difference was not statistically significant (p=0.085; **Supplementary Figure S2B**).

Finally, we examined the association between glucose metabolism markers and OS or PFS using a multivariate logistic regression analysis to evaluate each variable. A HOMA-beta ≥ 64.24 was an independent factor associated with prolonged OS in patients receiving ICI treatment after adjusting for age, sex, BMI, and eGFR (**Table 3**). Furthermore, HOMA-beta ≥ 66.43 was identified as an independent factor contributing to prolonged PFS in patients receiving ICI treatment (**Table 4**).

**Table 3:** Cox proportional-hazards regression: univariate and multivariate analyses of overall survival (OS).

	Univariate analysis			Multivariate analysis		
	HR	95% CI	P-value	HR	95% CI	P-value
Age	1.018	0.998–1.039	0.084	1.009	0.988-1.031	0.382
Male	1.187	0.747–1.888	0.468	1.196	0.747-1.916	0.456
BMI ≥ 18.58 kg/m^2^	0.465	0.294–0.736	0.001	0.481	0.299-0.772	0.002
HOMA-beta ≥ 64.24	0.581	0.372–0.909	0.017	0.623	0.393-0.989	0.045
eGFR (mL/min/1.73m^2^)	1.000	0.988–1.012	0.992	0.995	0.983-1.007	0.445

Multivariate analysis: independent variables are age, sex (male), BMI ≥18.58 kg/m^2^, HOMA-beta≥64.24, and eGFR. BMI, body mass index; eGFR, estimated glomerular filtration rate; HR, hazard ratio; CI, confidence interval.

**Table 4:** Cox proportional-hazards regression: univariate and multivariate analyses of progression-free survival (PFS).

	Univariate analysis			Multivariate analysis		
	HR	95% CI	P-value	HR	95% CI	P-value
Age	0.993	0.974-1.013	0.514	0.984	0.963-1.005	0.133
Male	0.890	0.550-1.440	0.636	0.857	0.523-1.405	0.541
BMI ≥ 18.44 kg/m^2^	0.693	0.435-1.103	0.122	0.661	0.408-1.068	0.091
HOMA-beta ≥ 66.43	0.612	0.380-0.987	0.044	0.557	0.339-0.916	0.021
eGFR (mL/min/1.73m^2^)	1.001	0.990-1.013	0.803	0.997	0.985-1.009	0.643

Multivariate analysis: independent variables are age, sex (male), BMI ≥18.44 kg/m^2^, HOMA-beta≥66.43, and eGFR. BMI, body mass index; eGFR, estimated glomerular filtration rate; HR, hazard ratio; CI, confidence interval.

## Discussion

In this study, we demonstrated that ICI administration induces fluctuations in glucose metabolism markers even in non-diabetic conditions. Notably, HOMA-beta was significantly higher in patients with OS ≥7 months than in those with OS <7 months, both before and 1 month after ICI administration. This work represents a novel contribution as it is the first to establish an association between HOMA-beta and prolonged OS in the context of ICI therapy, irrespective of the occurrence of diabetes.

The programmed death-1 (PD-1)/programmed death-ligand 1 (PD-L1) pathway plays a significant role in maintaining immunological homeostasis in the pancreas (10, 11). Typically, the expression of PD-L1 in pancreatic beta-cells is minimal. However, in the presence of inflammation, the upregulation of PD-L1 is induced by cytokines such as interferon. The findings of previous *in vitro* investigations conducted on non-diabetic human islets demonstrated that interferon-gamma (IFN-γ) plays a role in promoting PD-L1 in pancreatic beta-cells (12, 13). The precise mechanism by which ICIs cause harm to pancreatic beta-cell remains unclear. However, it has been hypothesized to impede inhibitory signals and stimulate T lymphocytes, leading to an increase in the number of CD8+ T cells. This increase in CD8+ T cells may result in the impairment of pancreatic cells and a subsequent decline in pancreatic endocrine function (14–16). However, considering the substantial contribution of local and systemic inflammation to the advancement of malignant tumors and its role in cancer progression and the survival of patients with cancer, it is plausible to suggest that the inflammation-induced PD-1/PD-L1 pathway might play a role in the development of pancreatic endocrine dysfunction (17, 18).

Numerous studies have documented a correlation between the effectiveness of ICI administration and the occurrence of irAEs (19). While the number of patients with CPI-DM is insufficient to make definitive conclusions, it is noteworthy that a significant number of these individuals have exhibited either partial or total antitumor response (20, 21). However, additional research is required to determine whether the occurrence of CPI-DM is linked to improved clinical outcomes. C-peptide serves as a marker of endogenous insulin in individuals who exhibit normal glucose tolerance, while HOMA-beta is utilized as an indication of beta-cell activity (22). In this study, we showed an association between an effectiveness of ICI and insulin secretion capacity. Notably, patients with HOMA-beta value ≥ 65 exhibited a statistically significant increase in OS after ICI administration. In general, hyperinsulinemia is known to influence the development of certain types of cancer, but no reports have been found to support the idea that cancer progression other than pancreatic cancer affects beta-cell function (23). On the other hand, cancer cachexia, which is observed in advanced cancer, causes weight loss with changes in body composition due to reduced caloric intake, chronic inflammation, and abnormal endocrine function. In glucose metabolism, decreased insulin secretion associated with decreased caloric intake and increased hepatic glucose production and hepatic glycogenesis lead to increased insulin resistance (24). However, the effect of advanced cancer on beta-cell function remains unclear. It has been postulated that the impact on the capacity for insulin secretion might indicate the effectiveness of ICI in the treatment of patients with cancer.

The use of ICI has emerged as a promising and efficacious approach for combating cancer across a diverse range of malignancies (1). Several factors influence the efficacy of ICI therapy (25). Hence, it is crucial to ascertain the determinants affecting the effectiveness of ICI therapy. PD-L1 is the initial predictive biomarker approved by the Food and Drug Administration (FDA) for ICI administration in individuals diagnosed with non-small cell lung cancer. Microsatellite instability, or deficient mismatch repair, is the second biomarker that has received FDA approval for the therapeutic management of solid tumors that are either unresectable or metastasized. Additionally, the utilization of tumor mutation burden data has received approval from the FDA for the therapeutic management of solid tumors that are either unresectable or metastasized (26). Furthermore, various patient characteristics, including sex, age, smoking history, and BMI, have been identified in the literature as potential predictors of treatment response to ICI (27–30). Several predictive variables have been documented in the context of ICI therapy. However, a clear predictive factor has not yet been established. Cortellini et al. reported that BMI serves as a predictive indicator for ICI treatment (31). In this study, we found that both HOMA-beta and BMI were significant prognostic indicators. The results regarding the association between BMI and OS were consistent with previous literature. For PFS, however, only HOMA-beta was a predictive factor associated with prolonged PFS. A significant association between BMI and PFS may be found by increasing the number of cases.

Despite these novel findings, this study has certain limitations. First, it had a single-center, prospective, observational design, and limited sample size. Conducting multicenter studies with larger sample sizes is necessary to validate our findings. Second, it is important to ascertain the generalizability of our findings to other forms of cancer, given the inherent limitations associated with focusing only on the specific malignancy examined in this study. Third, HOMA-beta is a rough estimate of endogenous insulin secretory capacity, but it is extremely stable in fasting and non-diabetic studies and is a frequently used test to evaluate beta-cell function (32). Additionally, owing to the inherent characteristics of the condition, the lack of a comprehensive evaluation of beta-cell activity prior to ICI administration makes it impossible to accurately determine the effects of ICI on beta-cells.

In summary, our study findings demonstrate an association between HOMA-beta and extended OS in the context of ICI therapy. Additionally, our work posits that the beta-cell activity prior to ICI administration may potentially serve as an indicator of the effectiveness of ICI therapy in individuals diagnosed with cancer. Future investigations should aim to provide a more comprehensive understanding of the biological mechanisms underlying these findings.

## Data Availability

The raw data supporting the conclusions of this article will be made available by the authors, without undue reservation.

## Supplementary material

Supplementary Figure 1 ROC curve analysis of the ability of BMI to predict the extended overall survival (OS) **(A)** and progression-free survival (PFS) **(B)**.

Supplementary Figure 2
**(A)** Kaplan–Meier curves of overall survival (OS) for patients with BMI ≥ 18.58 kg/m^2^ and < 18.58 kg/m^2^. **(B)** Kaplan–Meier curves of progression-free survival (PFS) for patients with BMI ≥ 18.44 kg/m^2^ and < 18.44 kg/m^2^.
