# Supplementary figures and images for: HOMA-beta independently predicts survival in patients with advanced cancer on treatment with immune checkpoint inhibitors
Source: Front Endocrinol (Lausanne). 2024 Dec 11;15:1439705. doi: 10.3389/fendo.2024.1439705 (PMC11668593; doi:10.3389/fendo.2024.1439705)

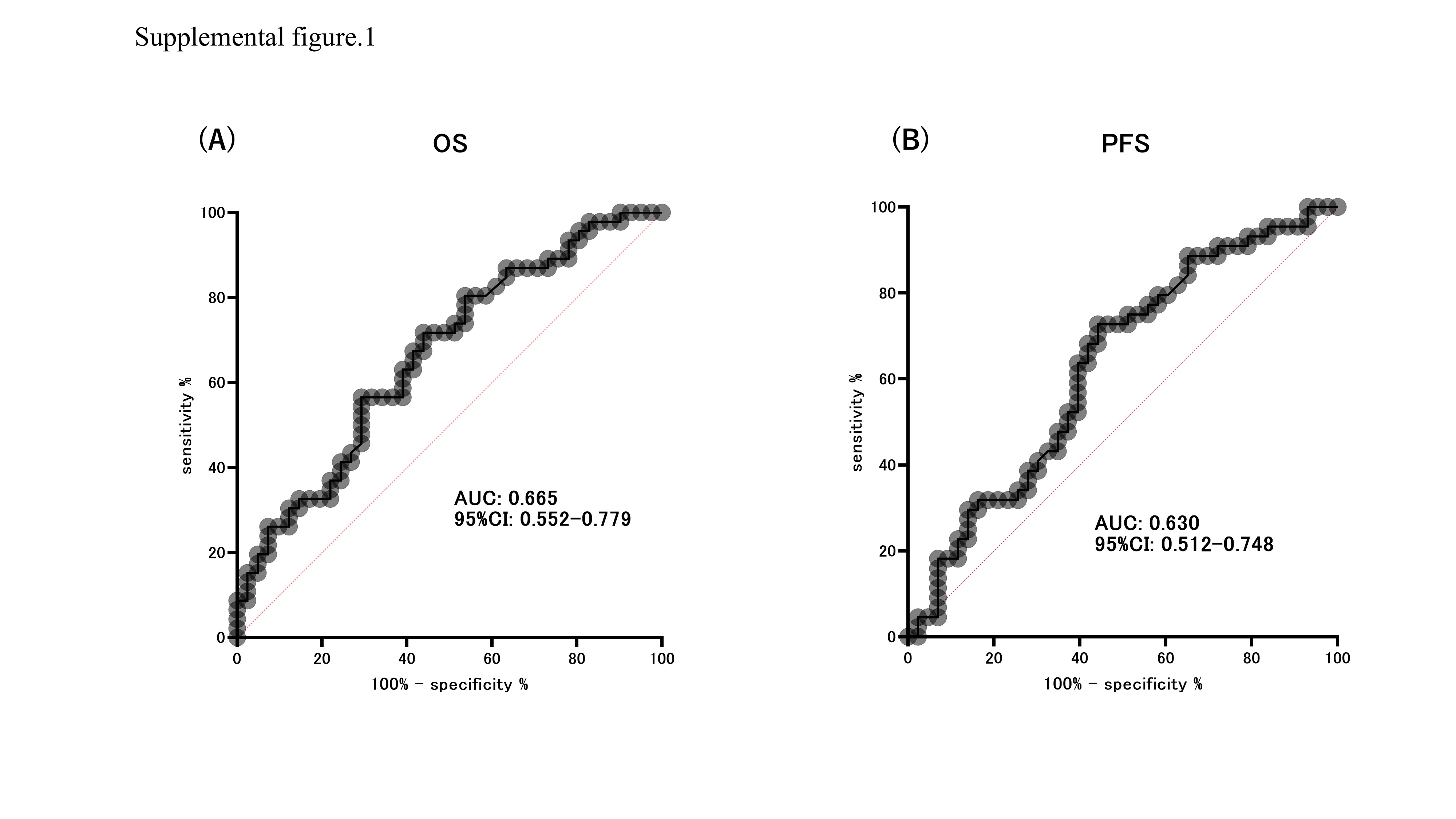

Supplement: Supplementary Figure 1 — ROC curve analysis of the ability of BMI to predict the extended overall survival (OS) (A) and progression-free survival (PFS) (B). [file Image1.jpeg]

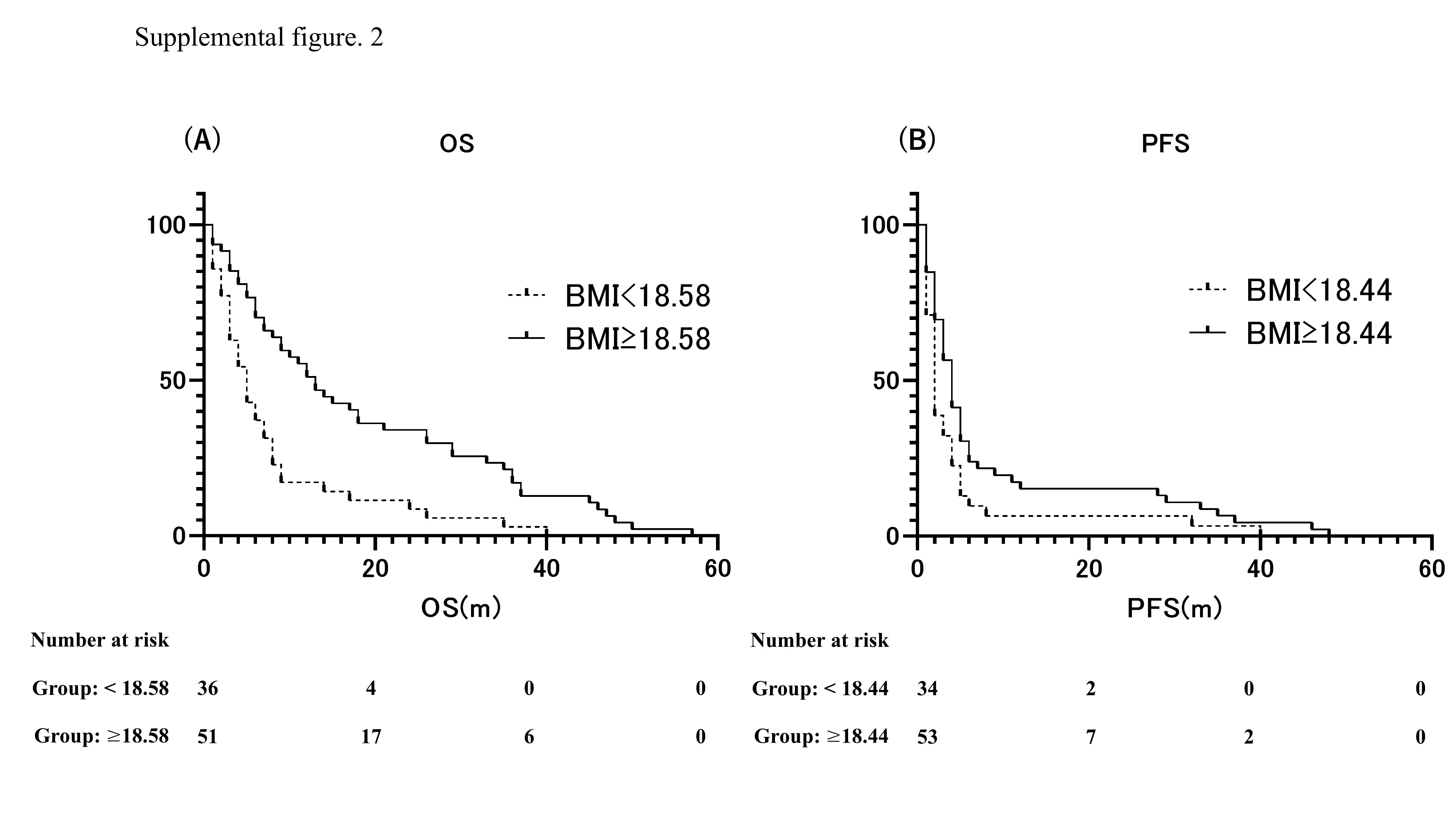

Supplement: Supplementary Figure 2 — (A) Kaplan–Meier curves of overall survival (OS) for patients with BMI ≥ 18.58 kg/m2 and < 18.58 kg/m2. (B) Kaplan–Meier curves of progression-free survival (PFS) for patients with BMI ≥ 18.44 kg/m2 and < 18.44 kg/m2. [file Image2.jpeg]
